# CCDC6 and USP7 expression levels suggest novel treatment options in high-grade urothelial bladder cancer

**DOI:** 10.1186/s13046-019-1087-1

**Published:** 2019-02-20

**Authors:** Francesco Morra, Francesco Merolla, Daniela Criscuolo, Luigi Insabato, Riccardo Giannella, Gennaro Ilardi, Aniello Cerrato, Roberta Visconti, Stefania Staibano, Angela Celetti

**Affiliations:** 1 Institute for the Experimental Endocrinology and Oncology, Research National Council, CNR, Naples, Italy; 2 Department of Medicine and Health Sciences “V. Tiberio”, University of Molise, Campobasso, Italy; 3 Department of Advanced Biomedical Sciences, University “Federico II”, Naples, Italy; 4 Urology Surgery Unit, A.Cardarelli Hospital, Naples, Italy

**Keywords:** Biomarkers, DNA damage, DNA repair, PARP-inhibitor, P5091, RRx-001, Viral mimicry, Epigenetic, Immunotherapy, Precision medicine

## Abstract

**Background:**

The muscle invasive form of urothelial bladder cancer (UBC) is a deadly disease. Currently, the therapeutic approach of UBC is mostly based on surgery and standard chemotherapy.

Biomarkers to establish appropriate drugs usage are missing. Deficiency of the tumor suppressor CCDC6 determines PARP-inhibitor sensitivity. The CCDC6 levels are modulated by the deubiquitinase USP7. In this work we scored CCDC6 and USP7 expression levels in primary UBC and we evaluated the expression levels of CCDC6 in correlation with the effects of the PARP-inhibitors combined with the USP7 inhibitor, P5091, in vitro. Since PARP-inhibitors could be enhanced by conventional chemotherapy or DNA damage inducers, we tested the new agent RRx-001, able to induce DNA damage, to prove the benefit of combined treatments in bladder cancer cells.

**Methods:**

The J82, T24, 5637 and KU-19-19 bladder cancer cells were exposed to USP7 inhibitor P5091 in presence of cycloheximide to analyse the CCDC6 stability. Upon the CCDC6 degradation induced by P5091, the cells sensitivity to PARP-inhibitor was evaluated by cell viability assays. The ability of the DNA damage inducer RRx-001 to modulate CCDC6 protein levels and H2AX phosphorylation was detected at immunoblot. The combination of USP7 inhibitor plus RRx-001 enhanced the PARP-inhibitor sensitivity, as evaluated by cell viability assays. The results of the scores and correlation of CCDC6 and USP7 expression levels obtained by UBC primary biopsies staining were used to cluster patients by a K-mean cluster analysis.

**Results:**

P5091 determining CCDC6 degradation promoted bladder cancer cells sensitivity to PARP-inhibitor drugs. RRx-001, by inducing DNA damage, enhanced the effects of the combined treatment. The immunohistochemical staining of both CCDC6 and USP7 proteins allowed to cluster the high grade (G3) UBC patients, on the basis of CCDC6 expression levels.

**Conclusions:**

In high grade UBC the identification of two clusters of patients based on CCDC6 and USP7 expession can possibly indicate the use of PARP-inhibitor drugs, in combination with USP7 inhibitor in addition to the DNA damage inducer RRx-001, that also acts as an immunomodulatory agent, offering novel therapeutic strategy for personalized medicine in bladder cancer patients.

**Electronic supplementary material:**

The online version of this article (10.1186/s13046-019-1087-1) contains supplementary material, which is available to authorized users.

## Introduction

Urothelial bladder cancer (UBC) is the most common cancer of the genitourinary tract and the ninth most common cancer worldwide [1]. Treatment of disease that presents localized to the urothelium is primarily surgical, systemic chemotherapy and radiotherapy mostly playing supporting roles. However, for patients with locally advanced or metastatic UBC (mUBC), the mainstay of treatment is systemic chemotherapy, and the paradigm has not changed or improved the outcomes for decades with a median overall survival of approximately 15 months [2].

Since 2016, systemic approaches using various immunotherapeutic strategies, with drugs targeting immune checkpoints, have revolutionized the treatment of several solid tumors including the mUBC [3–5], which demonstrates a unique immunogenicity profile from early to late stages of the disease.

Additionally, the association of antitumor immune checkpoint therapies with epigenetic agents is emerging as novel strategy to improve the power of the treatments also reducing side effects. The epigenetic agents unsilence epigenetically repressed viral genes present in the tumor, thus inducing an immune response and contributing to the anticancer activity [6, 7].

Recently, a novel first-in-class epigenetic and immunomodulator agent, RRx-001, has been developed [8]. RRx-001 induces apoptosis, determines the release of reactive oxygen- and nitrogen-species, activates the DNA damage response via ATM/H2AX phosphorylation, and decreases the DNA methytransferase (DNMT) levels and global methylation [8, 9]. The deubiquitylating enzyme USP7 is known to stimulate the DNMT1 activity, and conversely, USP7-siRNA reduces DNMT1 activity and decreases tumor cell viability [10]. Interestingly, RRx-001 plus USP7 inhibitor P5091 has been reported to trigger synergistic anti-tumoral activity, in multiple myeloma and different preclinical models [11]. Besides DNMT1, USP7 controls the turnover of further substrates, including the CCDC6 gene product [12]. CCDC6 encodes for a pro-apoptotic protein, often inactivated in thyroid and lung cancer upon fusion with various oncogenes [13–15]; CCDC6 is also impaired by somatic mutations, detected at low frequency in several tumors including bladder cancer (http://www.cbioportal.org/), as well as by its increased downregulation due to altered tuning of its modifying enzymes, the Fbxw7 E3 ubiquitin ligase and the USP7 deubiquitinase [12]. In response to genotoxic and oxidative stress, CCDC6 is phosphorylated by ATM kinase and involved in DNA double-strand breaks (DSBs) repair by homologous recombination (HR) [13]. Thus, the CCDC6 deficiency, accompanied by the HR-DNA repair defects, determines sensitivity to PARP-inhibitors, in lung, colon and prostate cancer cells, as recently demonstrated [12, 16, 17].

In this investigation our aim has been to analyse CCDC6 and USP7 expression levels in a series of primary urothelial bladder cancer, arranged in a Tissue Micro Array (TMA), by immunohistochemistry. Then, we have compared the CCDC6/USP7 expression scores to the tumor grade. Additionally, in a series of in vitro bladder cancer cells we have investigated whether the pharmacological inhibition of USP7, by lowering the levels of CCDC6, was able to impair the DNA repair processes by homologous recombination (HR), favouring the bladder cancer cells sensitivity to PARP-inhibitors. In the same cells, the antitumoral activity of the epigenetic agent RRx-001 in presence of the USP7 inhibitor P5091, and in combination with PARP-inhibitor drugs, has been investigated.

## Methods

### Cell lines, drugs and chemicals

The J82, T24, 5637, KU-19-19 bladder carcinoma cell lines were obtained by the “American Type Culture Collection” (ATCC), and cultured in the DMEM plus 10% of fetal bovine serum (Gibco, Paisley, UK). Olaparib (AZD2281) and P5091 were provided by SelleckChem. Cycloheximide, 5’Azacytidine were from SIGMA-Aldrich, Inc.; RRx-001 was from Med Chem.

### Sensivity test and design for drug combination

Antiproliferative activity was determined by a modified 3-(4,5-dimethylthiazole-2-yl)-2–5- diphenyltetrazolium bromide assay, CellTiter 96 AQueous One Solution assay (Promega), as 50% inhibitory concentration (IC50) values, according to manufacturer instructions.

Briefly, cells were plated in quintuplicate in 96-well plates at a density of 1000 cells per well, and continuously exposed to each drug for 144 h. Each assay was performed in quintuplicate and IC50 values were expressed as mean ± standard deviation. The results of the combined treatment were analyzed according to the method of Chou and Talaly by using the CompuSyn software program. The resulting combination index (CI) is a quantitative measure of the degree of interaction between different drugs. A CI value of unity denotes additive activity while CI > 1 denotes antagonism, and CI < 1 denotes synergy between agents [18].

### Western blotting and antibodies

Western blotting was performed as described [19, 20]. Immunoblotting experiments were carried out according to standard procedures and visualized using the ECL chemiluminescence system (Amersham/ Pharmacia Biotech).

The following antibodies were utilized: anti-CCDC6 (Abcam), anti-USP7 (Bethyl), anti-tubulin, (SIGMA-Aldrich, Inc), anti-PCNA (Millipore), anti-DNMT1 (Cell Signaling), anti-gH2AX (#05636) was from Millipore. Secondary antibodies were from Biorad, California.

### Plasmids and transfection

PcDNA4ToA-Myc-CCDC6, DR-GFP reporter, pGACGS I-SceI and CCDC6 shRNA (pLKO.1 puro) plasmids were transiently transfected in J82 cells with FuGene HD (Promega). CCDC6 shRNA pool and the pool of nontargeting vectors (ShCTRL) were from Sigma-Aldrich. The DR-GFP reporter plasmid is based on a construct developed by M. Jasin and contains two mutated GFP genes separated by a puromycin drug selection marker.

### HR assay

J82 cells were plated in a 60 mm plate and transfected with the DR-GFP reporter alone (as negative control), or together with the I-SceI gene. Wild type GFP was used as control for transfection efficiency. After 48 h cells were collected and analyzed by FACS analysis with BD Accuri C6 Flow Cytometer (BD Bioscience, Canada).

### Real time PCR

PCR reactions were performed on RNA isolated from cell lines using RNeasy Mini Kit (Qiagen) and reverse-transcribed using MuLVRT (Invitrogen). qRT-PCR analysis was performed with Syber Green (Agilent). Primer sequences are reported in the Supplementary section. To calculate the relative expression levels we used the 2-ΔΔCT method.

### TMA and IHC

Two pathologists (LI and FM) reviewed the whole routine hematoxylin-eosin (H&E) sections of 46 bladder samples to confirm the original diagnosis and to mark the most representative tumor areas useful for the TMA construction. For all selected cases, the tumour area for TMA construction was identified on the same paraffin donor blocks under the guidance of the corresponding previously marked H&E section and punched by a semiautomatic tissue-array instrument. Two cores for each tumor were taken. The tissue cores (3 mm in diameter) were carefully transferred to the recipient paraffin blocks. The filled recipient blocks were then placed on a metal base mold. The paraffin-embedding was then carried-out, by heating the blocks at 42 °C for 10 min and flattening their surface by pressing a clean glass slide on them. 4-μm sections were cut from each TMA using an ordinary microtome. The first section was stained with H&E to confirm the presence of tumor and the integrity of tissues. The other sections were mounted on a super frost slide (Microm, Walldorf, Germany) for the immunohistochemical evaluation of CCDC6 and USP7 proteins.

For CCDC6 and USP7 IHC assay, manual staining was performed as described [12, 21, 22].

### Statistical analysis

Statistical analysis was performed using SPSS software (IBM Corp. Released 2013. IBM SPSS Statistics for Windows, Version 22.0. Armonk, NY: IBM Corp.). For tissue biomarker expression correlation a Spearman correlation test was performed. A K.mean cluster analysis was performed to sort out to relatively homogeneous groups of cases based on selected characteristics (CCDC6 and USP7 IHC expression).

### Slide scanning and digital assessment

Slides scanning was performed with Leica Aperio AT2 scanner at 40x using embedded autofocusing methods.

Digital assessment has been executed with QuPath open source software (https://qupath.github.io/). Analysis protocol started with TMA dearrayer, generating a grid fulfilled with the TMA map cores. “Simple tissue detection” function has been used to identify areas covered by tissue to separate them from the background. “Cell detection” function has been used to identify single cells based on hematoxylin counterstaining. “Feature selection” function has been used to calculate features based on whom classification algorithm was run. Then, QuPath algorithm “Positive cell detections” has been used to calculate CCDC6 and USP7 IHC positivity.

## Results

### USP7 inhibitor, P5091, by increasing the turnover of CCDC6, determined a defect in DNA repair mediated by homologous recombination and sensitized bladder cancer cells to PARP-inhibitors

**Fig. 1:**
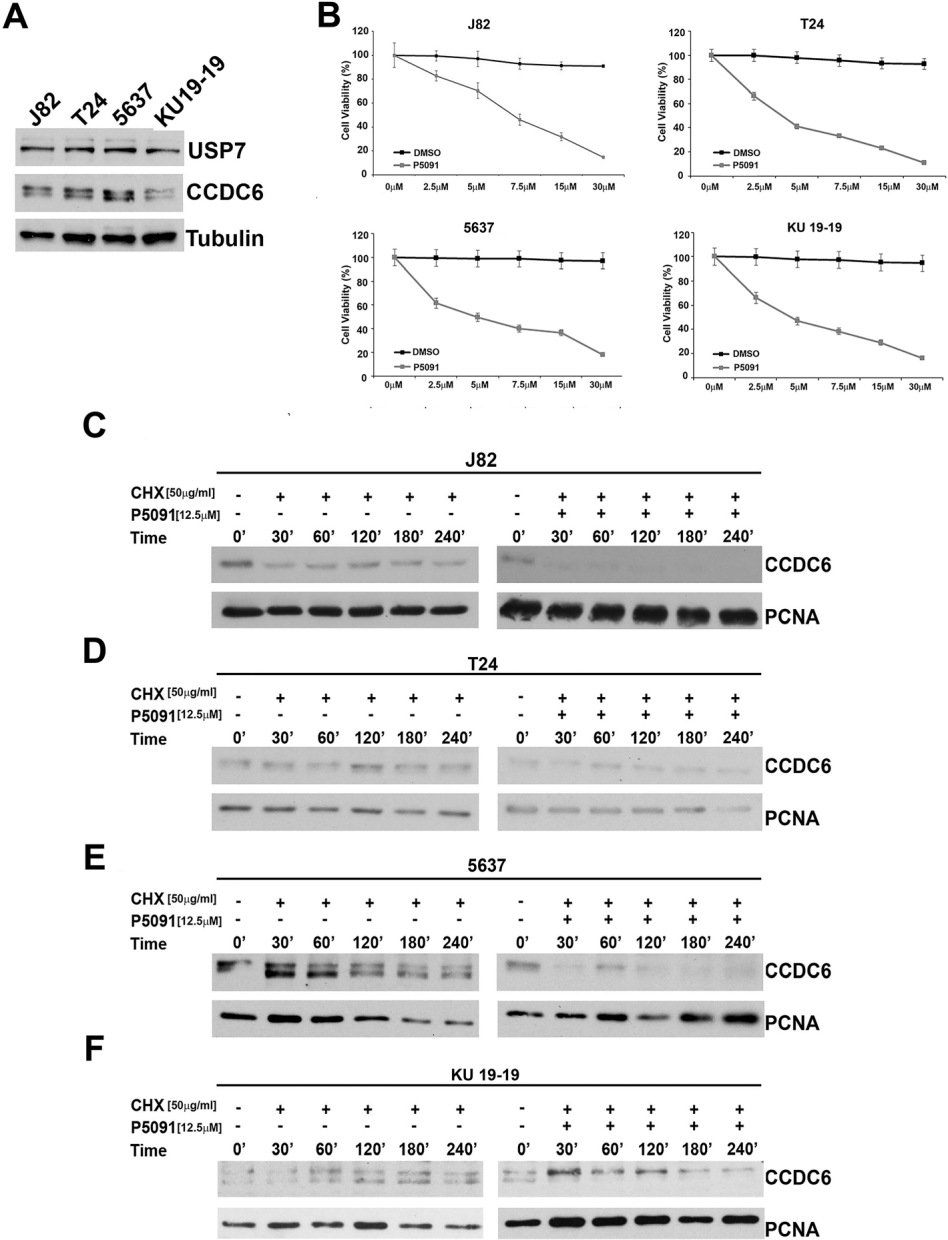
**a** Immunoblot analysis of CCDC6 and USP7 in the J82, T24, 5637, KU-19-19 bladder cancer cells. Anti-tubulin is shown as loading control. **b** P5091 exerts a cytotoxic effect on bladder cancer cells, J82 [7.9 μM IC50], T24 [4.21 μM IC50], 5637 [4.90 μM IC50] and KU-19-19 [4.83 μM IC50] . Cells were seeded in 96-well plates and 24 h later exposed to the vehicle (DMSO) or to P5091 at the indicated doses for 144 h. The viability of cells at 50% inhibitory concentration [IC50] value was analysed using a modified 3-(4,5-dimethylthiazole-2-yl)-2–5-diphenyltetrazolium bromide assay, CellTiter 96 Aqueous one Solution assay (Promega), The values are presented as mean standard deviation of three independent experiments. **c**, **d**, **e**, **f** J82, T24, 5637 and KU-19-19 cells were pretreated with P5091 [12.5 μM] for 4 h, or with DMSO, and exposed to cycloheximide (CHX) [50 μg/ml] for the indicated times. Total protein lysates were subjected to immunoblot analysis using anti-CCDC6 and anti-PCNA antibodies

The pharmacological inhibition of the deubiquitinase enzyme USP7 has shown antitumor properties in several tumor types [23] and also determined cytotoxic effects in a series of urothelial bladder cancer cells expressing appreciable levels of CCDC6 and USP7 proteins at western blot (Fig. 1a, b). In order to evaluate if the pharmacological inhibition of USP7 through P5091, by increasing the turnover of the CCDC6 protein, could influence the half-life of CCDC6, J82, T24, 5637 and KU-19-19 bladder cancer cells were treated with P5091 for 4 h, or with DMSO as a control, and then exposed to cycloheximide (CHX), to block new protein synthesis, at the indicated times.

**Fig. 2:**
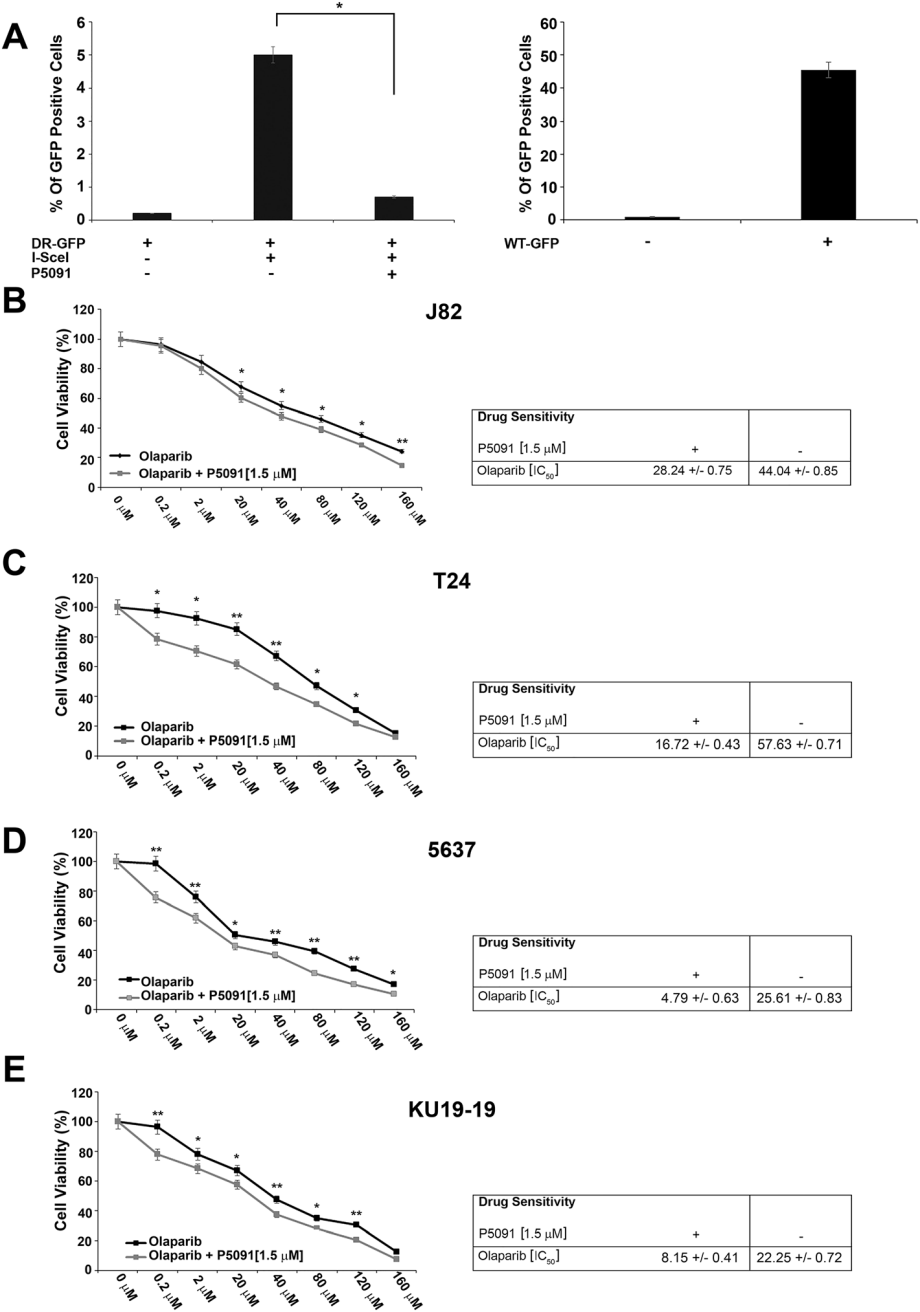
**a** J82 cells were pretreated with the vehicle (DMSO) or P5091 [8 μM] for 4 h and transfected with DR-GFP alone, as control, or together with I-SceI, for 48 h. The percentage of GFP positive cells, compared to controls, has been plotted on histograms representative of three independent experiments. Error bars indicate the standard error mean. Transfection efficacy has been plotted on the histograms shown on the right. **b-e** Survival fractions of J82, T24, 5637 and KU-19-19 cells treated with Olaparib, at the indicated doses, in presence or absence of P5091 [1.5 μM], for 144 h. Sensitivity to Olaparib, in presence or absence of P5091, was determined by a modified MTT assay (MTS), Cell Titer 96 AQueous One Solution assay, and expressed as IC50, i.e. the value that allows 50% of the inhibitory concentration. The IC50 values are expressed as mean ± the standard deviation. Statistical differences were determined by two-tailed Student’s t test. Statistical significance is displayed as: * *p* < 0.05; ** *p* < 0.01; *** *p* < 0.001

The immunoblot with anti-CCDC6 antibody indicated that the CCDC6 half-life was reduced upon P5091 pretreatment in bladder cancer cells (Fig. 1c, d, e, f). To verify whether the reduction of CCDC6 levels, induced by P5091, altered the double strand breaks (DSBs) DNA repair by homologous recombination (HR), the bladder cancer cells pretreated with P5091, or untreated, were transfected with the DR-GFP reporter plasmid alone, as a control, or together with the I-SceI plasmid, able to induce DSBs. The ability to repair the DSBs by HR was measured by flow cytometry and the frequency of HR was reported as a percentage of GFP positive cells. Treatment with USP7 inhibitor determined a significant decrease of the GFP positive cells, compared to non-treated cells, suggesting that the reduction of CCDC6 levels affected the DNA repair by HR in bladder cancer cells (Fig. 2a). Bladder cancer cells transfected with a wild type GFP plasmid were used as a control of transfection efficiency.

Since the HR defect is accompanied by increased sensitivity to PARP-inhibitors, in order to evaluate whether P5091, by lowering CCDC6, was able to sensitize the bladder cancer cells to the PARP-inhibitor Olaparib, bladder cancer cells were treated with different concentrations of Olaparib, in the presence or absence of P5091 and the cytotoxic effects of the treatment was quantified by a cell viability assay. We found that Olaparib, as single agent, showed a limited activity in reducing the viability of the bladder cancer cells while the addition of P5091 led to an increased sensitivity of the cells to Olaparib (Fig. 2b c, d, e).

### RRx-001 inhibited growth of bladder cancer cells

Recently, it has been reported that the USP7 inhibitor P5091 was able to act in association with a new antitumor agent, RRx-001. The association of the two drugs resulted in a pronounced reduction of the levels of the DNMT1 enzyme, responsible of the methylation pattern maintenance during DNA replication [11].

Several genomics studies have highlighted the importance of epigenetic changes and genomic instability in the development and progression of bladder cancer, suggesting, as a novel treatment option, the use of epigenetic drugs in association with immune checkpoint therapies [24, 25].

**Fig. 3:**
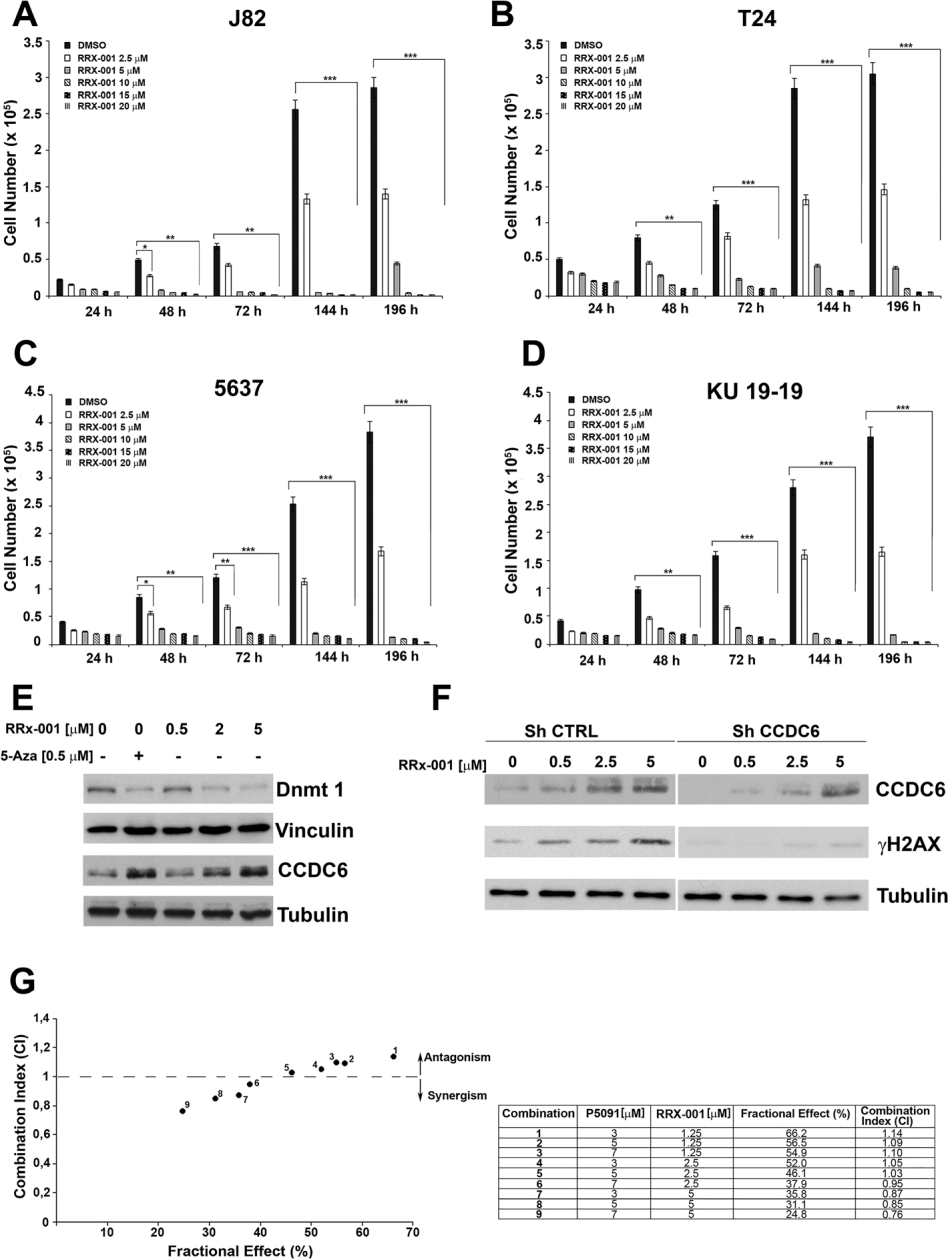
**a-d** J82, T24, 5637 and KU-19-19 cells were treated with different concentrations of RRx-001, or DMSO as control, and counted at the indicated times. The values represent the mean of three independent experiments +/− standard deviation. Statistical significance was verified by 2-tailed Student’s t-test (* *p* < 0.05; ** *p* < 0.01 and *** *p* < 0.001). **e** Protein levels of DNMT1 and CCDC6 in J82 cells treated with different concentrations of RRx-001, as determined by western blot. Vinculin and Tubulin were used as internal controls for sample loading. 5-AZA was used as positive control for inhibition of DNMT1 expression. **f** Whole cell lysates from J82 shCCDC6 or shCTRL cells, treated with different concentrations of RRx-001, or untreated, were immunoblotted with anti-CCDC6 antibody. γH2AX levels are shown. Tubulin was used as loading control. **g** J82 cells were treated with RRx-001, P5091 or RRx-001 plus P5091 for 144 h and then assessed for cells viability using a modified MTT assay (MTS), Cell Titer 96 AQueous One Solution assay. Isobologram analysis shows the synergistic antiproliferative activity of RRx-001 plus P5091 at the higher doses. In the left panel the graph derives from the values given in the table (right panel). In the graph the black dots below the dotted line indicate the presence of synergistic interaction between the two drugs. In the tables the Fractional Effect represent a percentage expression of the number of live cells. Values of CI < 1, CI = 1 and CI > 1 indicate respectively synergistic, additive and antagonistic effects

In order to evaluate whether RRx-001 was able to determine a cytotoxic activity in bladder tumor, bladder cancer cells were treated with different concentrations of RRx-001, or with DMSO as control, and counted at different times. The RRx-001 agent showed a cytotoxic effect in the bladder cancer cells at 48 h (Fig. 3a, b, c, d). Then, we treated the cells with RRx-001 at three different concentrations (0.5–2 - 5 μM) or with a pan-DNMT inhibitor, 5-AZA, at 0.5 μM, as a positive control, for 48 h, and as predicted, RRx-001 had the ability to negatively modulate DNMT1 levels (Fig. 3e).

Furthermore, the agent RRx-001, by downregulating the DNA-methyltransferase 1 (DNMT1) protein, generated in bladder cancer cells an immunomodulatory activity, by triggering an antiviral response in absence of a real viral infection - known as “viral mimicry” - through an interferon-mediated response (Additional file 1: Figure S1A-D) [7, 9, 26].

Interestingly, we observed that the RRx-001 agent induced CCDC6 protein stabilization, in a dose dependent manner (Fig. 3e). In fact, the RRx-001 agent, by exposing the bladder cancer cells to reactive oxygen species, can induce oxidative damage determining ATM phosphorylation and γH2AX activation [27]. However, by silencing CCDC6 in bladder cancer cells and upon treatment with low dose of RRx-001, we observed only a weak activation of γH2AX, compared to controls, that suggested cells tolerance to ROS, even if at the RRx-001 dose of 5 μM the γH2AX activation showed a feeble recover, regardless of the CCDC6 deficiency (Fig. 3f).

Recently, it has been reported that the combined treatment of P5091 and RRx-001 determined significant cytotoxicity in primary multiple myeloma cells [11]. Then, in order to evaluate the effects of the combined treatment of P5091 and RRx-001 in the bladder cancer cells, three fixed doses of P5091 (3–5 - 7 μM) and RRx-001 (1.25–2.5 - 5 μM) were utilized and viability assays were performed to quantify the cytotoxic effects of the treatments. We observed that the association of the two drugs at established concentrations showed a synergistic effect (CI < 1) in the bladder cancer cell lines (Fig. 3g).

### Combined treatment with P5091 and RRx-001 increased the sensitivity of bladder cancer cells to PARP-inhibitors

**Fig. 4:**
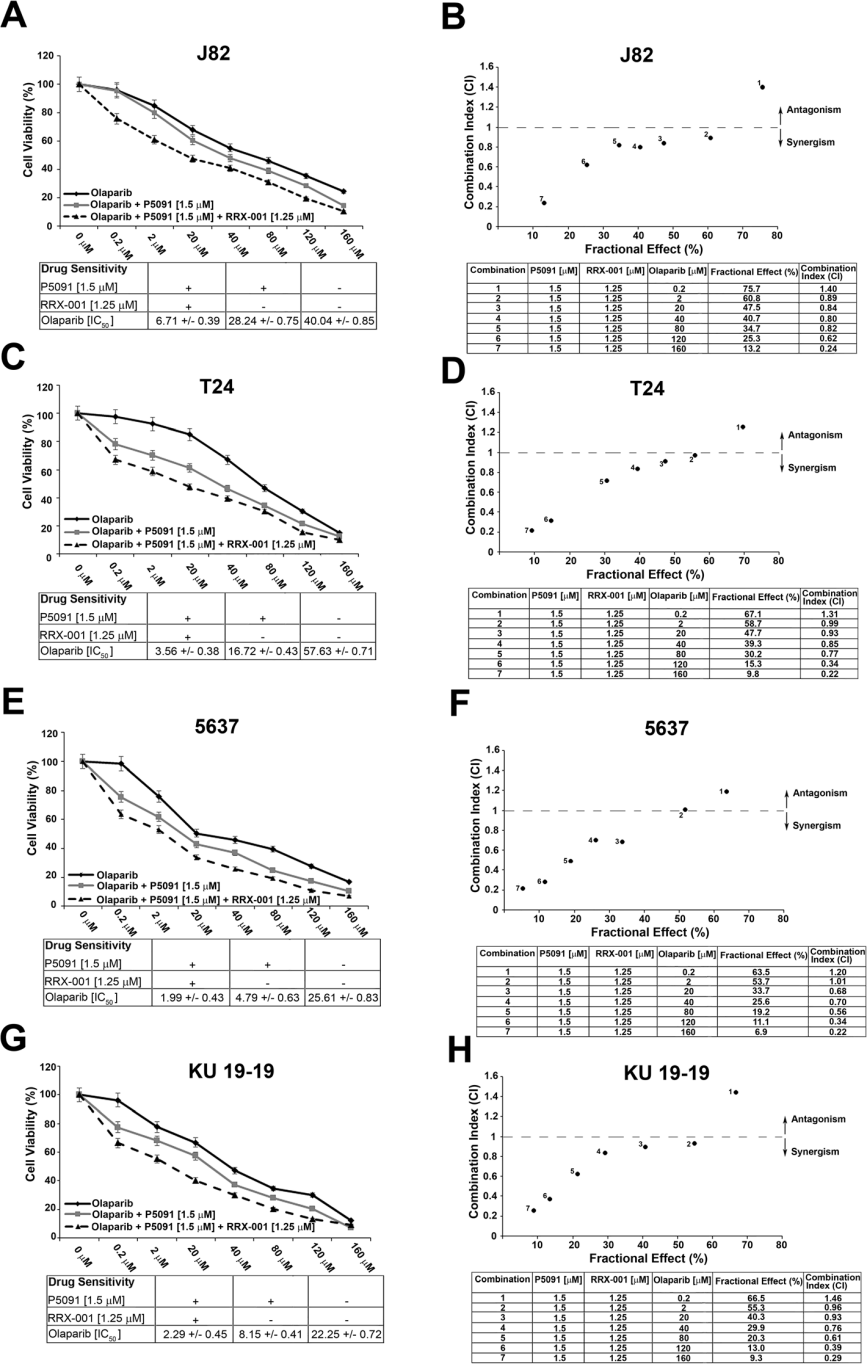
**a**, **c**, **e**, **g** Survival rates of J82, T24, 5637 and KU-19-19 cells treated with Olaparib, at the indicated doses, in presence or absence of P5091 [1.5 μM] and RRx-001 [1.25 μM], for 144 h. The sensitivity to the Olaparib, in presence or absence of P5091 [1.5 μM] and RRx-001 [1.25 μM], was determined by the modified MTT assay (MTS), Cell Titer 96 AQueous One Solution assay, and expressed as IC50, ie 50% of the inhibitory concentration. The values are expressed as mean ± the standard deviation. **b**, **d**, **f**, **h** Isobologram analysis show the synergistic antiproliferative activity of RRx-001 plus P5091 plus Olaparib (starting from the Olaparib concentration of [2 μM]). In the upper panels, the graphs derived from the values given in the tables (lower panels) are shown. In the graphs the black dots below the dotted line indicate the presence of synergistic interaction between the two drugs. In the tables the Fractional Effect represents a percentage expression of the number of live cells. Values of CI < 1, CI = 1 and CI > 1 indicate respectively synergistic, additive and antagonistic effects

**Fig. 5:**
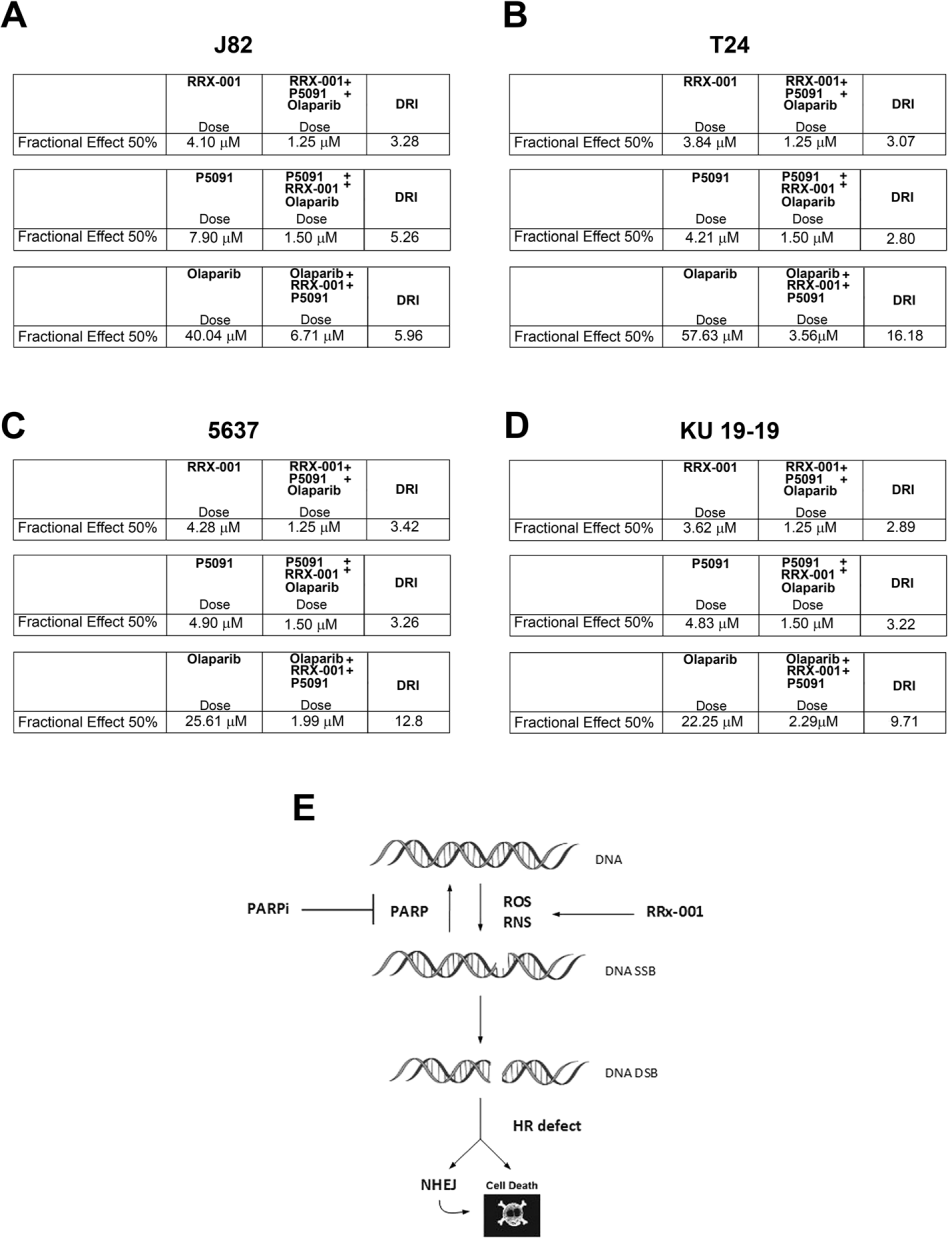
The Dose Reduction Index (DRI) for each drug reported in the table resulted > 1, as the dose to obtain the 50% Fractional Effect (IC50) resulted reduced upon the association of the three drugs: **a** J82, (RRx-001 + P5091 + Olaparib): RRx-001 [IC50 4.1 μM vs 1.25 μM]; P5091 [IC50 7.9 μM vs 1.5 μM]; Olaparib [IC50 40.04 μM vs 6.71 μM]; **b** T24, (RRx-001 + P5091 + Olaparib): RRx-001 [IC50 3.84 μM vs 1.25 μM]; P5091 [IC50 4.21 μM vs 1.5 μM]; Olaparib [IC50 57.63 μM vs 3.56 μM]; **c** 5637, (RRx-001 + P5091 + Olaparib): RRx-001 [IC50 4.28 μM vs 1.25 μM]; P5091 [IC50 4.90 μM vs 1.5 μM]; Olaparib [IC50 25.61 μM vs 1.99 μM]; **d** KU-19-19, (RRx-001 + P5091 + Olaparib): RRx-001 [IC50 3.62 μM vs 1.25 μM]; P5091 [IC50 4.83 μM vs 1.5 μM]; Olaparib [IC50 22.25 μM vs 2.29 μM]; **e** Sketch of RRx-001 contribution to DNA damage

In order to evaluate whether the new epigenetic agent RRx-001, that induce DNA damage through the release of reactive oxygen or nitrogen species, combined with P5091, that downregulates CCDC6 and impairs HR-DNA repair, might increase the sensitivity to PARPi in urothelial carcinoma cells, we treated the bladder cancer cells with different concentrations of the PARP-inhibitor Olaparib and with fixed doses of P5091 and RRx-001. The combination of P5091 and RRx-001, compared to the addition of P5091 alone, increased the sensitivity to the PARPi Olaparib [J82: Olaparib IC50 = 40.04 μM vs IC50 = 28.24 μM, in the presence of 1.5 μM of P5091, vs IC50 = 6.71 μM in the presence of 1.5 μM of P5091 and 1.25 μM of RRx-001] [T24: Olaparib IC50 = 57.63 μM vs IC50 = 16.72 μM, in the presence of 1.5 μM of P5091, vs IC50 = 3.56 μM in the presence of 1.5 μM of P5091 and 1.25 μM of RRx-001], [5637: Olaparib IC50 = 25.61 μM vs IC50 = 4.79 μM, in the presence of 1.5 μM of P5091, vs IC50 = 1.99 μM in the presence of 1.5 μM of P5091 and 1.25 μM of RRx-001], [KU-19-19: Olaparib IC50 = 22.25 μM vs IC50 = 8.15 μM, in the presence of 1.5 μM of P5091, vs IC50 = 2.29 μM in the presence of 1.5 μM of P5091 and 1.25 μM of RRx-001] (Fig. 4a, c, e, g). The results of the combined tretment seems to be extremely relevant when we analysed the Dose Reduction Index (DRI) that in clinical situation leads to reduced toxicity toward the host, while the therapeutic efficacy is retained [18]. Interestingly, by combining RRx-001, P5091 and Olaparib, we obtained a DRI > 1, that suggested a great dose reduction, with reduced toxicity while retaining the therapeutic effect (Fig. 5a, b, c, d). Finally, the association of the three drugs showed a synergistic effect (CI < 1) (Fig. 4b, d, f, h).

### CCDC6 and USP7 expression levels correlated in bladder cancer

**Fig. 6:**
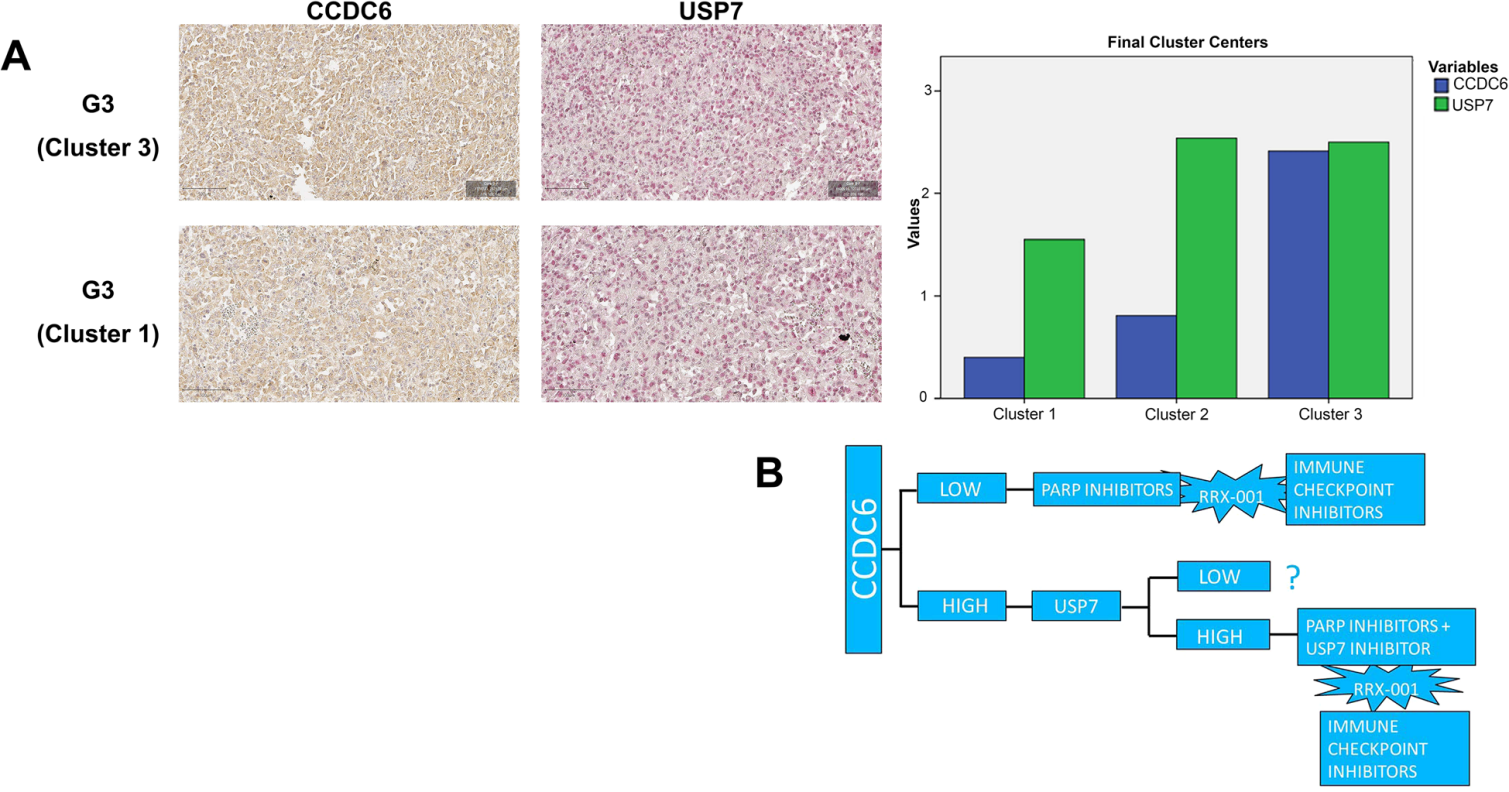
**a** Left: Representative images of G3 and G1 primary samples stained for CCDC6 and USP7 at immunohistochemistry. CCDC6 staining was revealed by DAB-conjugated secondary antibody, USP7 immunoreaction was revealed by Fast Red. The images show a high grade of concordance between CCDC6 and USP7 expression levels. Magnification, 40X. Right: K.mean cluster analysis showed two specific aggregations (cluster 1 and 3) with high prevalence of G3 tumor samples, showing two different patterns of biomarkers staining, and a third cluster (cluster 2) with an equal distribution between G1 and G3 tumors are shown. **b** CCDC6 e USP7 expression levels may suggest novel therapeutic scheme of personalized treatment in urothelial carcinoma

The CCDC6 and USP7 expression was evaluated semi-quantitively on 46 bladder cancer tumor samples; each tumor sample was present in duplicate in our TMA and analysed blindly by two pathologists (Fig. 6a, left).

The expression was graded as Negative (0), Low (1), Medium (2) and High (3), taking into accounts both the intensity of staining and the percentage of positive tumor cells. The final expression score was calculated as the average grade (Additional file 2: Figure S2A).

In order to test the significance of combined tissue IHC-expression of CCDC6 and USP7 we performed a non-parametric Spearman correlation test that proved to be extremely significant across all the tumor samples (Additional file 2: Figure S2B). We then compared the expression scores to the tumor grade.

In the first instance, we performed a cluster analysis in order to sort out those groups of samples homogeneous for CCDC6 and USP7 protein expression.

By performing a K-mean cluster analysis we identified specific aggregations among proteins’ expression levels across all our case series, and found 3 independent clusters according to the final centers (an analysis based on the z score, i.e. how many of the value standard deviation differs for the population mean value). By comparing the obtained clusters to the tumor grade, we observed two specific aggregations (clusters 1 and 3) with high prevalence of G3 tumor samples, showing two different patterns of biomarkers staining, and a third cluster (cluster 2) with an equal distribution between G1 and G3 tumors (Fig. 6a, right).

So, out of 46 bladder cancer tumor samples, we could identify a sub-group of high grade bladder cancers with a low expression of CCDC6 and a moderate expression of USP7 (cluster 1), and a sub-group of high grade tumors with higher expression of both the proteins (cluster 3). The third identified cluster grouped all the G1 samples and showed low levels of CCDC6 and high levels of USP7 (Fig. 6a; Additional file 4: Fig S4).

By this approach, then, we could further stratify the G3 tumor category into two different groups based on the expression pattern of CCDC6 and USP7.

Furthermore, by evaluating the clinicopathological features of the study population, we stratified the tumor samples in muscle-invasive (MID) and non-muscle-invasive disease (NMID). The samples distribution was statistically significant (*p* = 0.010) in CCDC6 expressing tumors, while it was not in the CCDC6 negative tumors (*p* = 0.102). In CCDC6 positive tumors, the prevalence of the simultaneous high expression of USP7 was 62.5% (15/24) in MID; conversely, the prevalence of USP7 low expression was 54.5% (12/22) in NMID. These data suggest that more then 60% of MID could benefit of a combined therapeutic approach with USP7 inhibitors and PARP inhibitors (Fig. 6b).

## Discussion

Urothelial bladder cancer is the fifth most common cancer in the United States. Approximately 30% of newly diagnosed patients present with muscle invasive bladder cancer, and 50% of them will have poor prognosis, high rate of distant metastases and short survival. Unfortunately, the management and mortality of the UBC has not substantially changed in the last 30 years. The mainstay of treatment is systemic chemotherapy with multidrug platinum combinations [2]. For more than 25 years, no new agents were approved by the FDA. In 2016 the programmed death-ligand 1 inhibitors, atezolizumab and nivolumab, were approved by the FDA for UBC that has progressed during or after platinum-containing chemotherapy, evaluating the safety and efficacy of the drugs [3, 28]. However, only 20 to 30% of patients with metastatic UBC achieved a partial or complete response to checkpoint immunotherapy as no methods to predict response are available [29]. Recently, in the muscle invasive form of UBC, mutations in genes involved in DNA damage response and repair, such as TP53, ATM, ERCC2, and, recently, CCDC6, have been reported at variable percentage [30, 31] [http://www.cbioportal.org], suggesting chemosensitivity or the use of PARP-inhibitor drugs for this tumor. The inhibition of PARP enzymes as anticancer strategy has been established on the basis of the biological concept of synthetic lethality, for which two genomic events, innocuous individually, become lethal when occurring together. When PARP enzymes are pharmacologically inhibited, the DNA single strand breaks cannot be repaired and eventually progress to toxic double strand breaks (DSBs), that result to be lethal in cells that lack HR repair capacity or have lost DNA repair genes [32, 33].

PARP inhibition has not been explored yet as a therapeutic strategy in bladder cancer patients, who do not carry a BRCA-mutant or BRCA-like phenotype. However, even if biomarkers and mechanisms of PARP-inhibitor-induced cytotoxicity are still poorly defined, several clinical trials are under way (NCT 03375307), also including PARP-inhibitors as single drug or in combination [29].

In several tumors, loss of wild-type p53 or ATM function can sensitize cancer cells to Olaparib [34–36], as well as CCDC6 deficiency that affect the DSBs DNA repair by homologous recombination can sensitize the tumor cells to PARP-inhibitors, that act synergically with genotoxic agent [12, 16].

In our study, we reported that 30% of primary bladder carcinoma samples (*N* = 46) exhibited low or undetectable staining of the CCDC6 protein. The remaining 70% showed a high intensity of staining, with a cytosolic reinforce that might also suggest a CCDC6 inactivation (Merolla F et al., in preparation). At IHC analysis, the expression levels of CCDC6 were significantly correlated to the levels of its deubiquitinase USP7. Interestingly, the scores compared to the tumors grade allowed stratification of the high grade bladder cancer in two clusters, on the basis of CCDC6 expression levels, which may be target for personalized patients treatment, in the future.

Furthermore, in in vitro bladder cancer cell system, we observed that the pharmacological inhibition of the USP7 deubiquitinase enhanced CCDC6 degradation, and by altering the DNA repair mechanisms mediated by homologous recombination, sensitized the bladder tumor cells to the cytotoxic effect of PARP-inhibitors. Nevertheless, myc-tagged vector-induced reconstitution of CCDC6 levels attenuated the sensitivity to PARP-inhibitors in bladder cancer cells. Conversely, shRNA-mediated CCDC6 silencing enhanced Olaparib sensitivity (Additional file 3: Figure S3), mostly supporting the specificity of action of the USP7 inhibitor through CCDC6 as substrate in determining PARP-inhibitor sensitivity. The USP7 inhibitor P5091 has shown an antitumor activity in several cancer systems, such as in colorectal carcinoma through the destabilization of β-catenin [37], in chronic lymphocytic leukemia by activation of the p53/p21 signaling axis [38], and in lung neuroendocrine tumors and prostate cancer [17, 21], in association with PARPi, through the destabilization of CCDC6 [12]. Indeed, in bladder cancer cells P5091 by reducing CCDC6 half-life affected the DSBs DNA repair and determined PARP-inhibitor sensitivity. Additionally, preclinical evidence suggests that Olaparib can radiosensitize bladder cancer cells derived from muscle invasive bladder cancer [35]. The results presented here are in support of the use of PARP-inhibitors for the treatment of primary urothelial bladder carcinomas that exhibit low levels of CCDC6 protein, as detected in 30% of primary BC of our analysis. Nevertheless, in UC that present high levels of CCDC6, that correlated to the levels of its modifier USP7, the treatment with the USP7 inhibitor P5091 sensitized urothelial tumors to PARP-inhibitors.

It has been reported that the DNA damage inducer RRx-001 acts synergically with the USP7 inhibitor P5091 in Multiple Myeloma [11]. In bladder cancer cells with HR defects caused by CCDC6 depletion (native or induced upon USP7 inhibitor addition) we observed that RRx-001, by exposing cancer cells to reactive oxygen species, enhanced the sensitivity to PARP-inhibitor drugs, as supported by the DRI value (DRI > 1) (Fig. 5a-e).

Oxidative DNA damage has been envisaged as emerging mechanism of carcinogenesis in bladder cancer [35]. However, in bladder cancer cells CCDC6 deficiency might result synthetic lethal with PARP-inhibition and the addiction of RRx-001, that exposes bladder cancer cells to oxidative damage, might amplify these effects, in accordance with the Dose Reduction Index that we calculated for each of the drugs, obtaining a value greater then 1 (DRI > 1).

In our investigation we also reported that the RRx-001 agent, by downregulating the DNA-methyltransferase 1 (DNMT1) protein, generated in bladder cancer cells an immunomodulatory activity by inducing an interferon response through epigenetic induction of viral mimicry [7, 9, 26, 39]. Therefore, it is possible to hypothesize the use of the RRx-001 agent in combination with the immune checkpoint inhibitors, in the treatment of urothelial carcinomas, as for different tumors [3, 6]. Nevertheless, RRx-001 has been already reported to enhance tumor response to antitumor immune checkpoint therapies and inserted in several clinical trials, also reducing the side effects (NCT02452970, NCT020966354, NCT02489903) [6, 24, 40]. Indeed, as in 2017 another anti immune checkpoint drug, pembrolizumab, has been FDA approved for bladder cancer patients, the use of RRx-001 might improve the quality of response [41]. Interestingly, treatment with PARP-inhibitors as a single agent therapy (NCT03375307), or in association with the anti PD-L1 drug durvalumab (NCT02546661), an antitumor immune checkpoint drug, are in Phase II/Phase1b clinical trials, respectively, for bladder cancer [28, 41, 42]. Therefore, the possibility to combine the immune checkpoint inhibitors, the PARP inhibitors and the immunomodulator agents (i.e. RRx-001) in bladder cancer should be envisaged.

Finally, the identification of two G3 clusters, that allow the stratification of the high-grade bladder urothelial cancer on the basis of CCDC6 expression levels, could help to design personalized treatment by combining all these drugs (Fig. 6a, b).

## Conclusion

In conclusion, we believe that in high grade UBC the distribution in two clusters of CCDC6 and USP7 protein levels can possibly indicate the use of PARP-inhibitor drugs, in presence or absence of the USP7 inhibitors, and the addition of the agent RRx-001 combined with the anti immune checkpoint drugs might offer novel personalized therapeutic scheme to bladder cancer patients (Fig. 6b).

## Additional files

Additional file 1: **Figure S1.** The agent RRx-001, by downregulating the DNA-methyltransferase 1 (DNMT1) protein, generated in bladder cancer cells an immunomodulatory activity, by triggering an antiviral response in absence of a real viral infection - known as “viral mimicry” - through an interferon-mediated response (Additional file 1: Figure S1A-D) [5, 7, 24]., i.e. leading to increased levels of IFNIII, [IFN λ 1/3 (IL- 29 / IL-28B)] and consequent upregulation of interferon-induced genes (ISGs) (Additional file 1: Figure S1A-D). In the panel D the modulation of the levels of two endogenous retroviral elements (ERV) (MLT1C49 and MLT2B4) are showed in the J82 cells, to confirm that the mechanism by which RRx-001 induced an interferon mediated response depended on viral mimicry [5, 7, 24]. These data show that RRx-001 is able to trigger an immunomodulatory effect in bladder cancer cells, through the “viral mimicry” mechanism. **A**) Expression levels of IL28A and IL29 in response to RRx-001 or 5-AZA. The J82 cells were treated with RRx-001 (0.5 μM) or 5-AZA (0.5 μM), for 24 h, and were then kept in culture, in a drug-free medium, for 7 consecutive days. IL28A and IL29 levels were measured by qPCR. **B-C**) RRx-001 induction of interferon stimulated genes. J82 cells were treated for 24 h with the RRx-001 agent (0.5 μM) (**B**) or 5-AZA (0.5 μM), as a control (**C**), and were kept in culture, in a drug-free medium, for 4 weeks. The expression levels of the four selected interferon-induced genes (IRF7, ISG15, OASL and DDX58, selected on account of their involvement in the dsRNA recognition pathway) were measured by qPCR. As shown in the figure, following the transient treatment with RRx-001, the four genes modulated by the interferon showed elevated levels at 2 weeks from the exposure. Conversely, two of the four genes (ISG15 and DDX58) maintained an increased expression up to 3 weeks after treatment. These results demonstrate that transient treatment with the RRx-001 agent led to a high and sustained expression over time of the selected ISGs in bladder cancer cells. **D**) RRx-001 induction of two selected endogenous retroviral elements (ERVs). J82 cells were treated for 24 h with RRx-001 (0.5 μM) or 5-AZA (0.5 μM), as a control, and were kept in culture, in a drug-free medium, for 7 consecutive days. The mRNA levels of the two selected ERVs (MLT1C49 and MLT2B4) were measured by qPCR. Transient treatment with RRx-001, or 5-AZA, led to an increase in ERV levels, compared to untreated cells (DMSO), as shown in the histograms. In A B, C, D the statistical significance was determined by 2-tailed Student’s t-test and is reported as: * *p* < 0.05 and ** *p* < 0.01. (JPG 901 kb)

Additional file 2: **Figure S2. A**) The table shows a statistic summary of the assigned scores to CCDC6 and USP7 expression levels in the analysed samples. **B**) The 2-tailed Spearman Rank correlation test proved to be extremely significant across all the tumor samples. (JPG 608 kb)

Additional file 3: **Figure S3. A**) J82 cells transiently transfected with control shRNAs (shCTRL) or sh-CCDC6 plasmids were treated with Olaparib for 144 h and then assessed for cells viability using a modified MTT assay (MTS), Cell Titer 96 AQueous One Solution assay. The values are expressed as IC50, i.e. the value that allows 50% of the inhibitory concentration. The IC50 values are expressed as mean ± the standard deviation. CCDC6 protein depletion was assessed by the anti-CCDC6 antibody at Western Blot. **B**) J82 cells transiently transfected with empty vector (EV), or with myc-CCDC6 wild type (myc-CCDC6) were treated with Olaparib for 144 h and then assessed for cells viability using a modified MTT assay (MTS), Cell Titer 96 AQueous One Solution assay. The values are expressed as IC50, i.e. the value that allows 50% of the inhibitory concentration. The IC50 values are expressed as mean ± the standard deviation. CCDC6 protein expression was assessed by the anti-myc antibody at Western Blot. In A and B anti-tubulin immunoblots are shown as loading control. (JPG 925 kb)

Additional file 4: **Figure S4.** a) Contingency table showing the frequency distribution of CCDC6 intensity IHC staining variable, stratified by USP7 intensity IHC, cross tabulated against clinic-pathological features of study population (MID = muscle-invasive disease; NMID = non-muscle-invasive disease); b) Statistical analysis of frequency distribution shown in panel A, significance has been calculated with a chi square test. Distribution of CCDC6 negative samples was not significant (*p* = 0.102). Distribution of CCDC6 expressing samples proved to be statistically significant (*p* = 0.010). (JPG 387 kb)

## Abbreviations

5-AZA: 5-azacytidine
ATM: Ataxia telengiectasia mutated
CCDC6: Coiled coil domain containing 6
CHX: Cycloeximide
CI: Combination index
DNMT1: DNA Methyl transferase 1
DRI: Dose reduction index
DSBs: Double strand breaks
H&E: Hematoxylin and eosin stain
IHC: Immunohistochemistry
mUBC: Metastatic UBC
PARP: Poly (ADP-ribose) polymerase DNMT
ROS: Reactive Oxygen Species
TMA: Tissue micro array
UBC: Urothelial bladder cancer
