# Supplementary figures and images for: CCDC6 and USP7 expression levels suggest novel treatment options in high-grade urothelial bladder cancer
Source: J Exp Clin Cancer Res. 2019 Feb 20;38:90. doi: 10.1186/s13046-019-1087-1 (PMC6381716; doi:10.1186/s13046-019-1087-1)

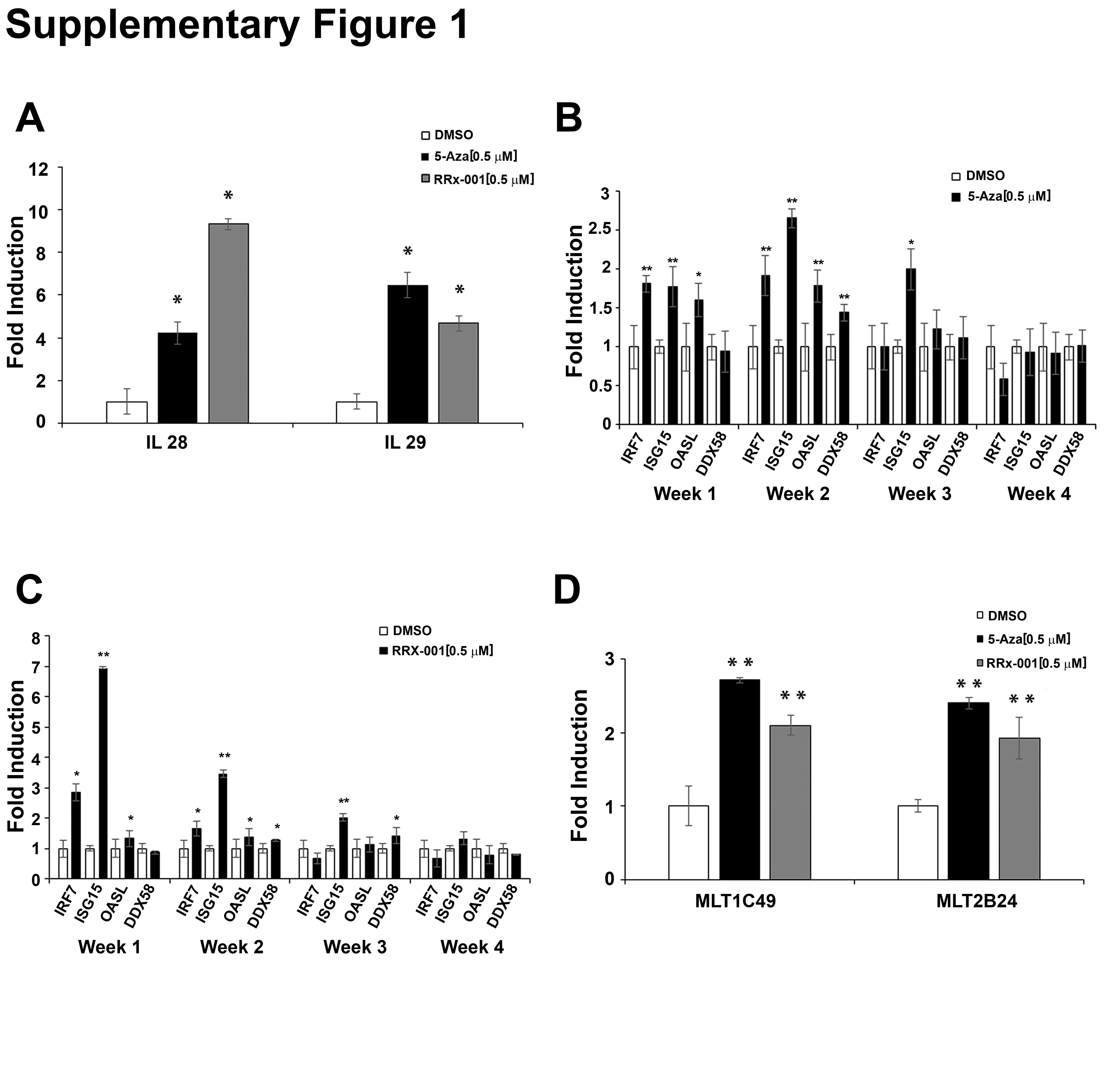

Supplement: Supplementary file 1 — Figure S1. The agent RRx-001, by downregulating the DNA-methyltransferase 1 (DNMT1) protein, generated in bladder cancer cells an immunomodulatory activity, by triggering an antiviral response in absence of a real viral infection - known as “viral mimicry” - through an interferon-mediated response (Additional file 1: Figure S1A-D) [5, 7, 24]., i.e. leading to increased levels of IFNIII, [IFN λ 1/3 (IL- 29 / IL-28B)] and consequent upregulation of interferon-induced genes (ISGs) (Additional file 1: Figure S1A-D). In the panel D the modulation of the levels of two endogenous retroviral elements (ERV) (MLT1C49 and MLT2B4) are showed in the J82 cells, to confirm that the mechanism by which RRx-001 induced an interferon mediated response depended on viral mimicry [5, 7, 24]. These data show that RRx-001 is able to trigger an immunomodulatory effect in bladder cancer cells, through the “viral mimicry” mechanism. A) Expression levels of IL28A and IL29 in response to RRx-001 or 5-AZA. The J82 cells were treated with RRx-001 (0.5 μM) or 5-AZA (0.5 μM), for 24 h, and were then kept in culture, in a drug-free medium, for 7 consecutive days. IL28A and IL29 levels were measured by qPCR. B-C) RRx-001 induction of interferon stimulated genes. J82 cells were treated for 24 h with the RRx-001 agent (0.5 μM) (B) or 5-AZA (0.5 μM), as a control (C), and were kept in culture, in a drug-free medium, for 4 weeks. The expression levels of the four selected interferon-induced genes (IRF7, ISG15, OASL and DDX58, selected on account of their involvement in the dsRNA recognition pathway) were measured by qPCR. As shown in the figure, following the transient treatment with RRx-001, the four genes modulated by the interferon showed elevated levels at 2 weeks from the exposure. Conversely, two of the four genes (ISG15 and DDX58) maintained an increased expression up to 3 weeks after treatment. These results demonstrate that transient treatment with the RRx-001 agent led to a high and sustained e [file 13046_2019_1087_MOESM1_ESM.jpg]

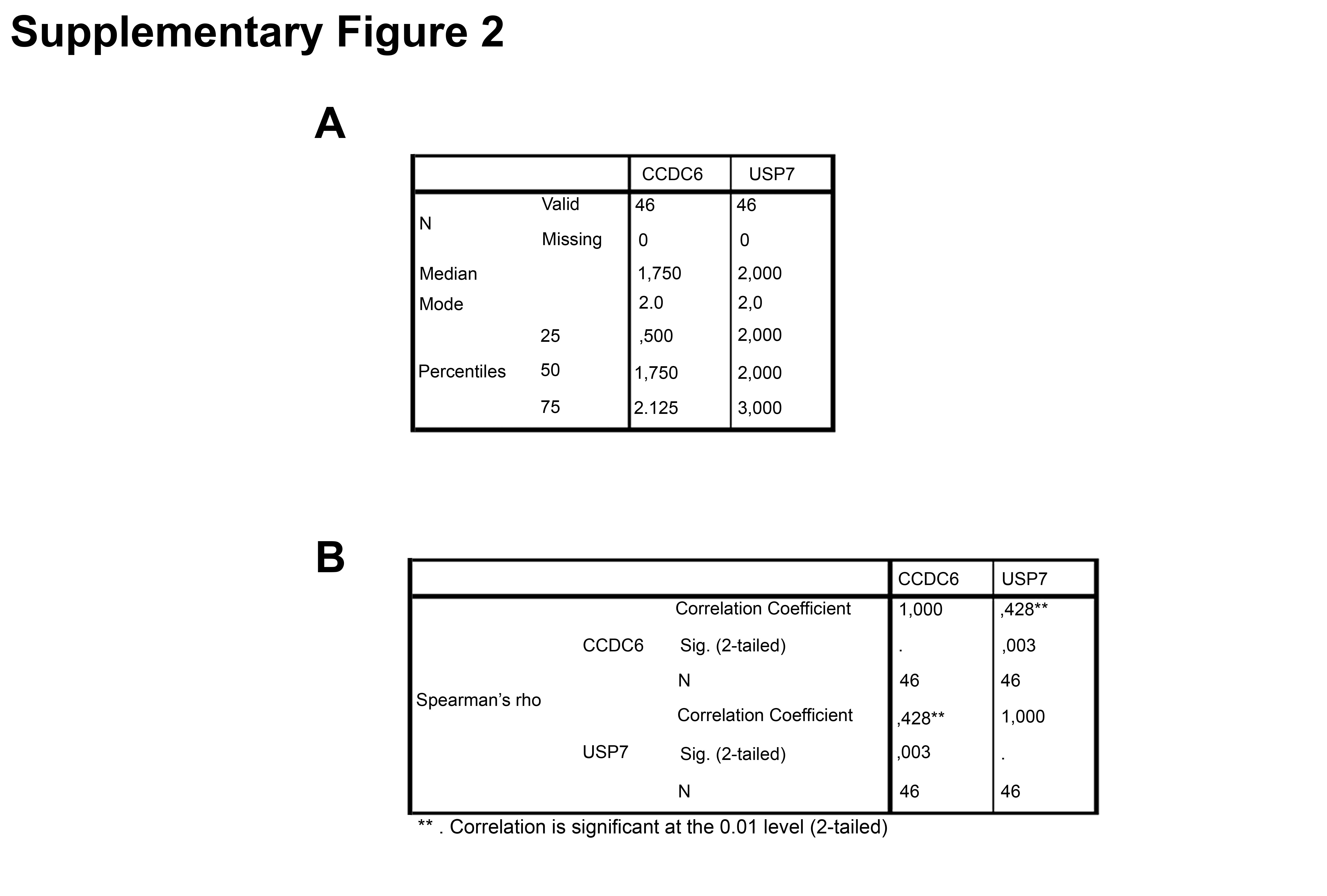

Supplement: Supplementary file 2 — Figure S2. A) The table shows a statistic summary of the assigned scores to CCDC6 and USP7 expression levels in the analysed samples. B) The 2-tailed Spearman Rank correlation test proved to be extremely significant across all the tumor samples. (JPG 608 kb) [file 13046_2019_1087_MOESM2_ESM.jpg]

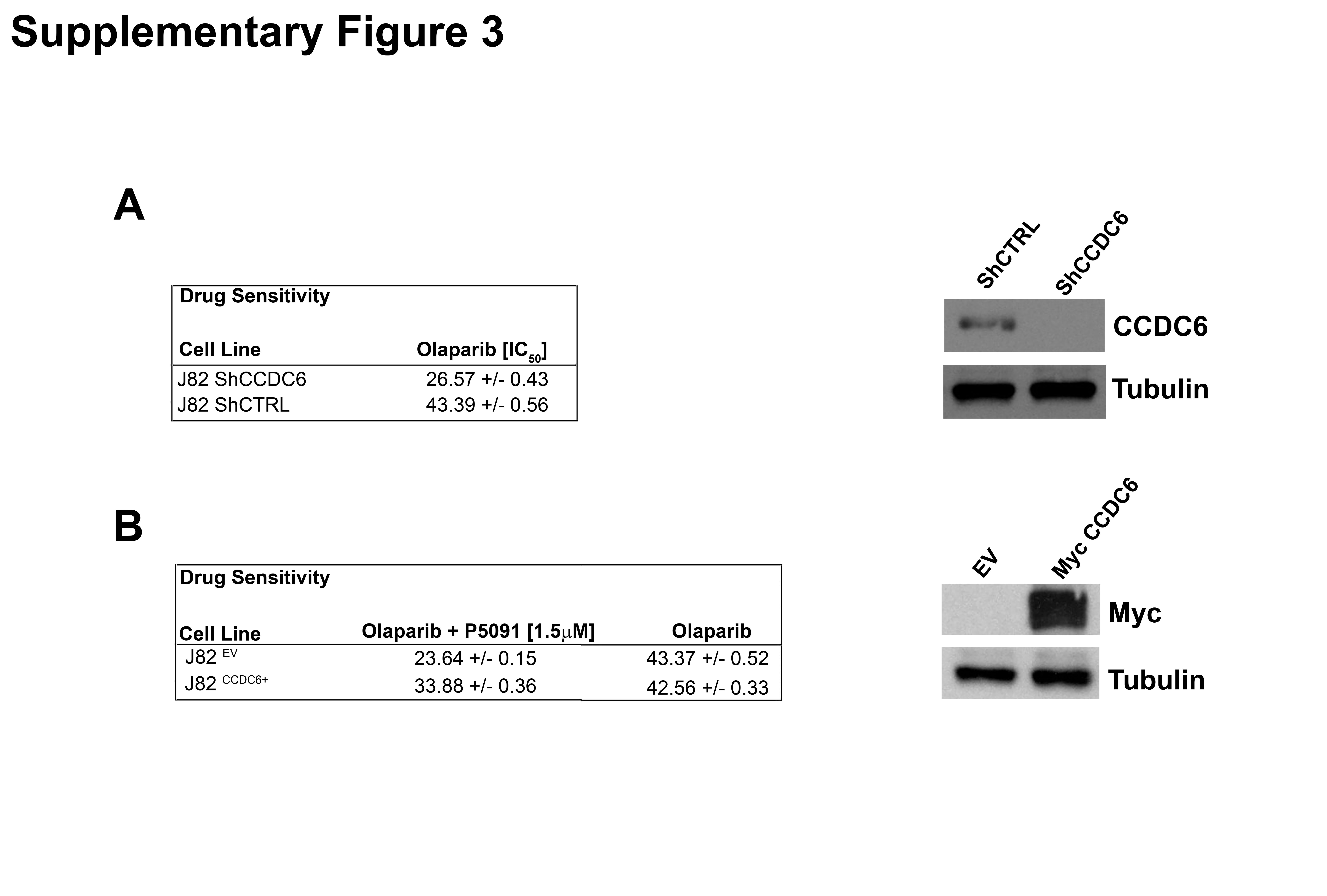

Supplement: Supplementary file 3 — Figure S3. A) J82 cells transiently transfected with control shRNAs (shCTRL) or sh-CCDC6 plasmids were treated with Olaparib for 144 h and then assessed for cells viability using a modified MTT assay (MTS), Cell Titer 96 AQueous One Solution assay. The values are expressed as IC50, i.e. the value that allows 50% of the inhibitory concentration. The IC50 values are expressed as mean ± the standard deviation. CCDC6 protein depletion was assessed by the anti-CCDC6 antibody at Western Blot. B) J82 cells transiently transfected with empty vector (EV), or with myc-CCDC6 wild type (myc-CCDC6) were treated with Olaparib for 144 h and then assessed for cells viability using a modified MTT assay (MTS), Cell Titer 96 AQueous One Solution assay. The values are expressed as IC50, i.e. the value that allows 50% of the inhibitory concentration. The IC50 values are expressed as mean ± the standard deviation. CCDC6 protein expression was assessed by the anti-myc antibody at Western Blot. In A and B anti-tubulin immunoblots are shown as loading control. (JPG 925 kb) [file 13046_2019_1087_MOESM3_ESM.jpg]

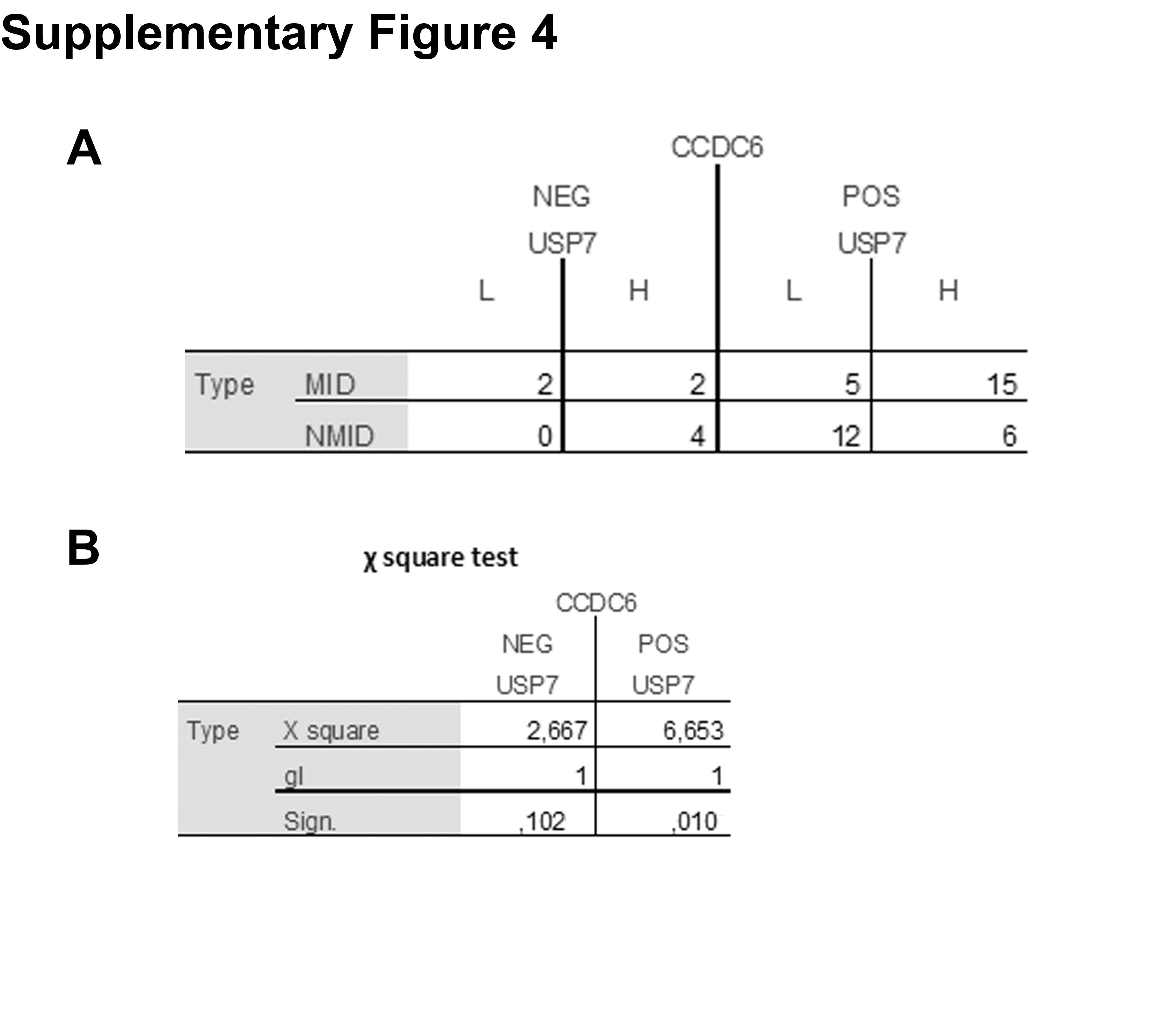

Supplement: Supplementary file 4 — Figure S4. a) Contingency table showing the frequency distribution of CCDC6 intensity IHC staining variable, stratified by USP7 intensity IHC, cross tabulated against clinic-pathological features of study population (MID = muscle-invasive disease; NMID = non-muscle-invasive disease); b) Statistical analysis of frequency distribution shown in panel A, significance has been calculated with a chi square test. Distribution of CCDC6 negative samples was not significant (p = 0.102). Distribution of CCDC6 expressing samples proved to be statistically significant (p = 0.010). (JPG 387 kb) [file 13046_2019_1087_MOESM4_ESM.jpg]
